# Policy perception, job satisfaction and intentions to remain in rural area: evidence from the National Compulsory Service Programme in China

**DOI:** 10.1186/s41256-024-00348-z

**Published:** 2024-04-30

**Authors:** Yanrong He, Peicheng Wang, Yanrong Du, Hange Li, Yanhua Chen, Jiming Zhu

**Affiliations:** 1 Vanke School of Public Health, Tsinghua University, Beijing, 100084 China; 2 School of Medicine, Tsinghua University, Beijing, China; 3 Institute for Healthy China, Tsinghua University, Beijing, China

**Keywords:** Rural areas, GPs, Retention, Perception towards the policy, Job satisfaction

## Abstract

**Background:**

Exploring factors that may influence general practitioners (GPs)’ intentions to remain in rural area is necessary to inform the training and placement of future medical workforce in rural area. However, little is known about how GPs’ perception towards the National Compulsory Service Programme (NCSP) and job satisfaction impact their turnover intention. This paper explores GPs’ intentions to remain in rural China and how their policy perception and job satisfaction predict the intentions.

**Methods:**

We conducted a cross-sectional, online survey from December 2021 to February 2022 to investigate GPs’ perception towards NCSP, job satisfaction, and intentions to remain in rural area. Eligible participants were GPs who were required to provide health services as part of NCSP at township health centres of 9 provinces which could represent all NCSP GPs in China. Multinomial logistic regression analyses were performed to explore the associations between policy perceptions, job satisfaction, and intentions to remain.

**Results:**

Of 3615 GPs included in the analysis, 442 (12.2%) would like to remain in rural area and 1266 (35.0%) were unsure. Results of the multinomial logistic regression analyses showed that compared with GPs who would leave, GPs with higher perception scores for the restriction on taking postgraduate exam (RRR: 1.93, 95% CI 1.72, 2.16) and the commitment to work for six years (RRR: 1.53, 95% CI 1.31, 1.78) were more likely to remain. In contrast, GPs who had higher perception scores for completing standardised residency training (RRR: 0.75, 95% CI 0.64, 0.88) and passing National Medical Licensing Examinations (RRR: 0.74, 95% CI 0.62, 0.87) were more likely to leave. GPs who were satisfied with the freedom of choosing work methods (RRR: 1.52, 95% CI 1.25, 1.84) and chances of promotion (RRR: 1.60, 95% CI 1.32, 1.94) were more likely to remain.

**Conclusions:**

This study highlights the significance of policy perception and job satisfaction on GPs’ intentions to remain in rural area. Factors such as career advancement and the empowerment of GPs to build on and use their skills and abilities should be taken into account when designing rural placement programmes.

**Supplementary Information:**

The online version contains supplementary material available at 10.1186/s41256-024-00348-z.

## Background

The quantity, quality and distribution of health workforce has been widely recognized as a crucial determinant of health system performance [1]. The availability of a qualified and motivated health workforce contributes to the capacity of health systems to deliver services to the population [2]. However, nearly all countries, particularly rural areas were struggling with the shortage and maldistribution of health workforce that may hamper the progress towards the Sustainable Development Goals and leads to inequalities in health outcomes [3]. In the United States and Canada, only 11.0% or less of physicians provide services in rural areas [4, 5]. The number of physicians per 1000 population in India is about 0.7 for rural communities, in contrast with 1.3 for urban areas [6]. As with other countries, China is confronting with great challenges in the imbalance and inequity in health workforce. In China, the amount of health workforce per 1000 was 1.5 times higher in urban than in rural areas in 2021[7].

As a critical intermediate level of a three-tier rural medical system in China, township health centres (THCs) has played a pivotal role in delivering rural medical care to hundreds of millions of people for more than forty years [8]. However, THCs often face understaffing issues due to the difficulties of recruiting health workforce and employing health workers with high education [9, 10]. In response to the inequalities of healthcare workforce availability between rural and urban areas, the National Development and Reform Commission and five other ministries jointly issued the National Compulsory Service Programme (NCSP) in March 2010 [11]. Twenty two provinces that suffered severe rural physician shortage in central and western China were targeted. Students with rural backgrounds were exclusively recruited and were subsidized to complete the 5-years undergraduate studies [12]. In return, students are obligated to provide rural health service as general practitioners (GPs) in designated THCs for 6 years, during which 3 years were required for the standardised residency training (SRT) [13]. The programme aims to train GPs who have passed the National Medical Licensing Examinations (NMLE) during the service period to meet the demands of health workforce in rural China [14]. In total, almost 60,000 students were recruited under this scheme between 2010 and 2020, and more than 10,000 of them had worked in the designated THCs [15].

Despite the great achievements NCSP has made in recruiting rural health workforce in the past few years, challenges still remain in GPs’ willingness to retain in rural communities [16]. For instance, the study conducted in Shannxi province found that 92.6% of 230 GPs who had enrolled in NCSP (NCSP GPs) expressed their intentions to break the contract after graduating from the programme [14]. In Xinjiang autonomous region, only 7.8% of 1200 NCSP GPs intended to remain after the compulsory service [17]. By contrast, the proportion of health practitioners who would like to remain after fulfilling the compulsory service was 53.0% in rural Australia [18]and 33.9% in rural Canada [19]. Turnover intention has been a major challenge for THCs in rural China and is associated with a variety of factors such as poor living conditions and infrastructure, and the unsustainable work environment [20]. Job satisfaction, as an indicator of the attitude and feelings an individual has about their work [21], has also been identified as a crucial factor in predicting turnover intention of rural health workforce [20, 22, 23].

While there have been a large body of investigations on turnover intention and job satisfaction among primary health workforce in rural China [20, 24–26], there is a research gap regarding GPs’ perceptions towards NCSP and its potential impact on their turnover intention in rural area. Given the unique role of NCSP GPs, it is necessary to investigate their perceptions towards such policies as the restriction on taking postgraduate exam [16]. Our study sought to examine the impacts of GPs’ perception towards NCSP and job satisfaction on their intentions to remain. Our findings can offer valuable suggestions to increase the retention of GPs in rural area and may provide guidance for other countries embarking on a similar trajectory.

## Methods

### Design, setting, and participants

We carried out a cross-sectional study between December 2021 and February 2022 to investigate GPs’ perception towards NCSP, job satisfaction, and intentions to remain in rural area. Based on the number of students enrolled by NCSP, we used stratified random sampling to select 9 provinces out of all the 22 provinces in central and western China where NCSP was implemented. The 9 provinces were Hainan, Hubei, Shanxi, Anhui, Guangxi, Guizhou, Qinghai, Xinjiang and Jilin. To ensure the representativeness and generalizability of the sample, the following criteria were met: (1) the number of GPs from the selected provinces was nearly one third of all NCSP GPs in the 22 provinces; (2) the selected provinces were located in four economic regions of China; (3) the economic development status of the 9 selected provinces measured by GDP could be comparable to that of the other 13 provinces that also implemented NCSP. All in all, our sample could represent all NCSP GPs in China. Details of these information can be found in Additional file 1: Table S1.

### Measures

#### Intentions to remain after contract expires

The primary outcome of this study was GPs’ intention to remain, evaluated by the question “Will you remain after the contract expires?” Responses included “yes”, “no” and “unsure”.

#### Socio-demographic characteristics

We asked the participants to specify their gender (male or female), age, place of living before joining NCSP (Urban or Rural), working hours per week (≤ 40, 41–48, 49–54, ≥ 55), monthly net income (≤ 3000, 3001–4000, 4001–5000, > 5000), marital status (married or single), years of service (1, 2, 3, ≥ 4), and presence of children (yes or no).

#### Perception towards NCSP

As is stipulated in the policy, seven items were used to measure GPs’ perceptions towards NCSP. The items were further classified into three categories. The category of Management of exit from the program (MEFP) included restrictions on taking postgraduate exam (MEFP-1), restrictions on changing designated rural settings (MEFP-2), and the commitment to work for six years (MEFP-3). Penalty for breaching the contract (PBC) included the refund of tuition and a payment for the fine (PBC-1), and a record in the integrity management (PBC-2). The other two items in the In-service training (IST) were the completement of SRT (IST-1) and a pass for NMLE (IST-2). The items were developed and issued by the National Development and Reform Commission with the aim of tackling with the shortage of health workforce in rural China [27]. Detailed descriptions of the seven items can be found in Table 1. All the items were measured using a five-point Likert scale (1 = “strongly disagree”, 2 = “disagree”, 3 = “neutral”, 4 = “agree” and 5 = “strongly agree”). Participants who scored higher than 3 were classified into the group of “Agree”. Participants who scored 3 were classified as “neutral”, and participants who scored lower than 3 were in the group of “Disagree”. Cronbach’s alpha of the seven items in our sample was 0.76, considered acceptable in reliability test.

**Table 1:** Descriptions and rationale of NCSP

NCSP	Descriptions	Rationale
MEFP-1: Restrictions on taking postgraduate exam	NCSP GPs were not allowed to take postgraduate exam	To decrease the possibility of GPs breaking the contract and leaving for urban areas
MEFP-2: Restrictions on changing designated rural settings	NCSP GPs were not allowed to change designated rural settings	To meet the urgent needs of THCs for health workforce
MEFP-3: Commit to work for six years	NCSP GPs were required to provide compulsory service in the designated THCs for six years	To be as an offset of the waived tuition and accommodation expenses during GPs’ undergraduate study, and to help tackle with the shortage of health professionals in rural THCs
PBC-1: Refund tuition and pay for the fine	If break the contract, NCSP GPs should refund waived tuition and compensate for the liquidated damages	To ensure the fulfillment of the contract
PBC-2: Record in the integrity management	Breach of the contract will be recorded in the physician integrity management	In February 2013, China launched a national policy that stipulated the establishment of an integrity system for health professionals [28]. GPs who breach the contract will be reported to the relevant national department by the end of the year. The information will be shared among health institutions and be used as evidence for GPs’ recruitment in public health institutions, residency training and postgraduate admissions [29]
IST-1: Complete the SRT	NCSP GPs should complete the three-year SRT during the six-year service period	SRT was first introduced in 2013 with China’s embarkment of a nationwide reform of medical education. The 3 years’ postgraduate medical education was to ensure a basic minimum of quality among doctors [30]
IST-2: Pass NMLE	NCSP GPs should obtain the health professional certificate during the service period	According to the law on Licensed Doctors in China, “The Doctors’ refers to medical workers who have obtained certificate as qualified doctors and are registered and employed in medical services, disease prevention or health-care institutions [31]”

#### Job satisfaction

We used a 10-item Warr-Cook-Wall (WCW) job satisfaction questionnaire to evaluate job satisfaction [32]. The questionnaire measures overall job satisfaction and satisfaction with nine aspects of work (physical work condition, freedom of working methods, colleagues and fellow workers, recognition for work, amount of responsibility, income, opportunity to use abilities, hours of work, and chance of promotion). Each aspect was rated on a five-point Likert scale (1 = “very unsatisfied”, 2 = “unsatisfied”, 3 = “neutral”, 4 = “satisfied”, 5 = “very satisfied”). Participants who scored higher than 3 were classified into the group of “Satisfied”. Participants who scored 3 were classified as “neutral”, and participants who scored lower than 3 were in the group of “Dissatisfied”. Cronbach’s alpha in our sample was 0.93.

### Data collection

All the THCs that had enrolled NCSP GPs within the mandate of provincial-level Health Commissions were informed to encourage their NCSP GPs to participate in the investigation. GPs who were working in the THCs and had been enrolled in NCSP were invited. An internet-based self-administered questionnaire was used for the investigation. The questionnaire was discussed and formulated by experts who already had abundant experience in doing NCSP research. To control the quality of the survey, we added rules to the answering of the survey. For instance, it might be possible that the designated THCs disseminated the survey to GPs who did not enrol in NCSP. We prevented this by asking the question at the beginning of the survey if participants had joined NCSP. If the answer is no, then exit the survey. The question on the province would help us exclude GPs who were not from the selected 9 provinces. Participants who had completed the questionnaire would receive a compensation as return. All participants had been fully informed of the purpose the study.

### Statistical analysis

Descriptive statistics were used to determine the socio-demographic characteristics of GPs, their perception towards the policy, job satisfaction, and their intentions to remain. Results were presented with frequencies and percentages for categorical variables, and with means and standard variation for continuous variables. Univariate analyses were performed to summarize the socio-demographic characteristics of the sample. Differences in scores of policy perception and job satisfaction among GPs who would remain, leave and were unsure were tested using Chi-Squared test. Multinomial logistic regression was used to determine the associations of policy perception and job satisfaction with GPs’ intentions to remain and unsure relative to those who would leave. Multinomial logistic regression is an extension of binary logistic regression and is appropriate for our three categorical outcome variables [33]. Relative risk ratio (RRR) and confidence intervals were calculated for each independent variable of GPs who would remain and were unsure compared with the reference group, GPs who would leave. RRR more than 1.0 indicated that with a one unit increase in the predictor variable, there is an increased possibility of being in the remain or unsure group compared with the leave group, after adjusting for all other key predictors and covariates. All statistical analyses were performed using Stata version 17.0 (Stata Corp LLC, College Station, Texas, USA). Statistical significance was set at P < 0.05.

## Results

### Sample description

In total, 3615 participants completed the survey with a response rate of 50.7% (3615/7135). The participants were NCSP GPs recruited between 2010 and 2013 who had already completed their five years’ undergraduate study, and had or would complete their six years’ obligatory services in the designated THCs at the time of the investigation. As is shown in Table 2, 52.8% of GPs had no intention to remain after contract expires and 35.0% were unsure. Details of the results by provinces can be found in Additional file 1: Table S2. The mean (SD) age of GPs was 28.3 (2.0) years; 53.1% were female. 90% of the GPs lived in rural area before being enrolled in NCSP. Nearly half of them (47.0%) were married, and 60.0% had children. Over one-third (33.3%) of the GPs worked more than 55 h per week, while only 16.7% of them earned more than 5000 CNY ($746) per month. 37.9% of the GPs served for one year, and 9.9% of them had worked more than four years.

**Table 2:** Socio-demographic characteristics of NCSP GPs (N, %)

Variable	Total	Intention to remain after contract expires		
		Yes	No	Unsure
Total	3615	442 (12.2)	1907 (52.8)	1266 (35.0)
*Gender*				
Male	1695 (46.9)	217 (12.8)	944 (55.7)	534 (31.5)
Female	1920 (53.1)	225 (11.7)	963 (50.2)	732 (38.1)
*Age, mean (SD), years*	*28.3 (2.0)*			
≤ 28	1991 (55.1)	232 (11.7)	1050 (52.7)	709 (35.6)
29–40	1624 (44.9)	210 (12.9)	857 (52.8)	557 (34.3)
*Place of living before joining NCSP*				
Urban	360 (10.0)	48 (13.3)	199 (55.3)	113 (31.4)
Rural	3255 (90.0)	394 (12.1)	1708 (52.5)	1513 (46.5)
*Working hours per week, mean (SD), hours*				
≤ 40	1143 (31.6)	161 (14.1)	529 (46.3)	453 (39.6)
41–48	915 (25.3)	116 (12.7)	464 (50.7)	335 (36.6)
49–54	354 (9.8)	38 (10.7)	200 (56.5)	116 (32.8)
≥ 55	1203 (33.3)	127 (10.6)	714 (59.4)	362 (30.1)
*Monthly net income, CNY*				
≤ 3000	821 (22.7)	130 (15.8)	438 (53.4)	253 (30.8)
3001–4000	1397 (38.6)	149 (10.7)	778 (55.7)	470 (33.6)
4001–5000	793 (21.9)	78 (9.8)	405 (51.1)	310 (39.1)
> 5000	604 (16.7)	85 (14.1)	286 (47.4)	233 (38.6)
*Marital status*				
Married	1700 (47.0)	212 (12.5)	934 (54.9)	554 (32.6)
Single	1915 (53.0)	230 (12.0)	973 (50.8)	712 (37.2)
*Years of service*				
1	1370 (37.9)	166 (12.1)	688 (50.2)	516 (37.7)
2	1139 (31.5)	132 (11.6)	617 (54.2)	390 (34.2)
3	747 (20.7)	83 (11.1)	417 (55.8)	247 (33.1)
≥ 4	359 (9.9)	61 (17.0)	185 (51.5)	113 (31.5)
*Presence of children*				
Yes	1020 (60.0)	130 (12.7)	569 (55.8)	321 (31.5)
No	680 (40.0)	82 (12.1)	365 (53.7)	233 (34.3)

Exchange rate during the study period: 1 USD = 6.35 CNY

### Scores of perception towards NCSP among GPs

Table 3 presents the scores of policy perception among NCSP GPs. Overall, there were significant differences in policy perception between GPs who would remain, leave and were unsure. Completing the SRT and passing NMLE had the equally highest score. Whereas, the restrictions on taking postgraduate exam had the lowest score when compared with other policy items. In other words, the number of GPs who disagreed with the restrictions on taking postgraduate exam was the largest when compared with those of other items. The number of GPs who agreed with completing the SRT and passing NMLE were higher than those of other items.

**Table 3:** Scores of perceptions towards NCSP among GPs

Items of NCSP	Score	GPs who agreed with the policy^a^ N (%)	Intentions after contract expires N (%)			*p*-Value
	Mean (SD)	Total N = 3615	Remain N = 442	Leave N = 1907	Unsure N = 1266	
IST-1: Complete the SRT	4.35 (0.83)	3146 (87.0)	361 (81.7)	1675 (87.8)	1110 (87.7)	0.002
IST-2: Pass NMLE	4.35 (0.80)	3169 (87.7)	375 (84.8)	1691 (88.7)	1103 (87.1)	0.067
PBC-1: Refund tuition and pay for the fine	3.22 (1.17)	1641 (45.4)	273 (61.8)	744 (39.0)	624 (49.3)	< 0.001
PBC-2: Record in the integrity management	2.95 (1.30)	1397 (38.6)	278 (62.9)	574 (30.1)	545 (43.0)	< 0.001
MEFP-2: Restrictions on changing designated rural settings	2.94 (1.28)	1316 (36.4)	260 (58.8)	553 (29.0)	503 (39.7)	< 0.001
MEFP-3: Commit to work for six years	2.88 (1.10)	976 (27.0)	223 (50.4)	352 (18.5)	401 (31.7)	< 0.001
MEFP-1: Restrictions on taking postgraduate exam	1.84 (1.07)	335 (9.3)	128 (29.0)	73 (3.8)	134 (10.6)	< 0.001

^a^GPs who scored higher than 3 were classified into the group of “Agree”

### Scores of job satisfaction among NCSP GPs

Table 4 presents the scores of job satisfaction among NCSP GPs. The score of the overall satisfaction was 2.78. Scores for all the nine items of job satisfaction had significant differences between GPs who would remain, leave and were unsure. NCSP GPs were less satisfied with their income and chance of promotion when compared with other aspects of the job. The number of GPs who were not satisfied with the income was the highest when compared with the number of other items. In contrast, colleagues and fellow workers had the highest score of job satisfaction among NCSP GPs.

**Table 4:** Scores of job satisfaction among NCSP GPs

Job satisfaction	Score	GPs who were satisfied ^a^	Intentions after contract expires N (%)			*p*-Value
	Mean (SD)	Total N = 3615	Remain N = 442	Leave N = 1907	Unsure N = 1266	
Colleagues and fellow workers	3.45 (0.99)	1795 (49.7)	331 (74.9)	704 (36.9)	760 (60.0)	< 0.001
Recognition for work	3.24 (0.98)	1379 (38.1)	295 (66.7)	501 (26.3)	583 (46.1)	< 0.001
Freedom of working method	2.99 (1.07)	1154 (31.9)	274 (62.0)	407 (21.3)	473 (37.4)	< 0.001
Amount of responsibility	2.91 (1.04)	996 (27.6)	250 (56.6)	292 (15.3)	424 (33.5)	< 0.001
Hours of work	2.86 (0.96)	755 (20.9)	208 (47.1)	240 (12.6)	307 (24.2)	< 0.001
Overall satisfaction	2.78 (0.98)	699 (19.3)	247 (55.9)	142 (7.4)	310 (24.5)	< 0.001
Opportunity to use abilities	2.69 (1.02)	644 (17.8)	223 (50.5)	149 (7.8)	272 (21.5)	< 0.001
Physical work condition	2.65 (1.08)	698 (19.3)	228 (51.6)	161 (8.4)	309 (24.4)	< 0.001
Chance of promotion	2.54 (1.06)	562 (15.5)	208 (47.1)	120 (6.3)	234 (18.5)	< 0.001
Income	2.28 (1.05)	385 (10.7)	169 (38.2)	71 (3.7)	145 (11.5)	< 0.001

^a^GPs who scored higher than 3 were classified into the group of “Satisfied”

### Association between perceptions towards policy, job satisfaction and intentions to remain

Table 5 presents the results of multinomial logistic regression analyses. In model1, GPs with the one unit increase in their perception towards the restriction on taking postgraduate exam had 1.93 times the risk of remaining in rural area versus leaving (RRR = 1.93, *P* < 0.001). However, GPs who had higher perceptions towards completing SRT and passing NMLE had a significantly decreased possibility of remaining in rural area versus leaving (RRR = 0.75 and 0.74 respectively, *P* < 0.001). In model 2, GPs who had higher satisfaction with income and chance of promotion showed a higher likelihood of remaining in rural area over leaving (RRR = 1.94 and 1.60 respectively, *P* < 0.001). The increased satisfaction with the freedom of working methods and opportunity to use abilities were also associated with an increased likelihood of remaining over leaving (RRR = 1.52 and 1.48 respectively, *P* < 0.001).

**Table 5:** Associations between perception towards NCSP, job satisfaction and intentions to remain after contract expires in multinomial logistic regression analysis

	Variables	Stay vs. Leave RRR (95% CI)	Unsure vs. Leave RRR (95% CI)
Model 1: Perception towards the policy	MEFP-1	1.93 (1.72, 2.16) ***	1.44 (1.32, 1.57) ***
	MEFP-2	1.37 (1.21, 1.55) ***	1.08 (1.01, 1.16) **
	MEFP-3	1.53 (1.31, 1.78) ***	1.32 (1.21, 1.45) ***
	PBC-1	0.94 (0.81, 1.09)	1.02 (0.94, 1.11)
	PBC-2	1.35 (1.18, 1.55) ***	1.11 (1.03, 1.20) ***
	IST-1	0.75 (0.64, 0.88) ***	0.94 (0.85, 1.04)
	IST-2	0.74 (0.62, 0.87) ***	0.87 (0.78, 0.96) ***
Model 2: Job satisfaction	Physical working condition	1.40 (1.18, 1.66) ***	1.27 (1.14, 1.40) ***
	Freedom of working method	1.52 (1.25, 1.84) ***	1.18 (1.05, 1.31) ***
	Colleagues and fellow workers	1.12 (0.90, 1.38)	1.30 (1.15, 1.47) ***
	Recognition for work	1.06 (0.84, 1.34)	0.97 (0.85, 1.11)
	Amount of responsibility	1.09 (0.89, 1.33)	1.04 (0.93, 1.16)
	Income	1.94 (1.65, 2.27) ***	1.37 (1.24, 1.51)***
	Opportunity to use abilities	1.48 (1.19, 1.83) ***	1.17 (1.04, 1.33) **
	Hours of work	0.74 (0.60, 0.90) ***	0.89 (0.79, 1.01) *
	Chance of promotion	1.60 (1.32, 1.94) ***	1.31 (1.17, 1.47) ***

Model 1 examined the association between perception towards the policy and intentions to remain after contract expires, adjusting for all the socio-demographic factors; Model 2 examined the association between job satisfaction and intentions to remain after contract expires, adjusting for all the socio-demographic factors. Statistically significant at *p < 0.1; **p < 0.05; ***p < 0.01

*MEFP-1* Restrictions on postgraduate study, *MEFP-2* Restrictions on changing designated rural settings, *MEFP-3* Commit to work for six years, *PBC-1* Refund tuition and pay for the fine, *PBC-2* Record in the integrity management, *IST-1* Complete the SRT, *IST-2* Pass NMLE

## Discussion

Based on the large representative sample of NCSP GPs in China, we investigated GPs’ perception towards NCSP and job satisfaction, and identified the factors that were associated with their intentions to remain in rural area. Overall, only 12.2% of GPs expressed an intention to remain. The intention to stay was influenced by GPs’ perceptions towards the policy and job satisfaction. Our findings offered multiple implications for future educational programming and policy schemes to address the shortage of healthcare workforce in rural area.

Compared to other items of NCSP, the restriction on taking postgraduate exam had the largest number of GPs who reported a low score of perception. But those with higher scores for the perception of this policy are more likely to remain in rural area. The finding was consistent with another study conducted in Sichuan province, which also identified pursuing a master’s degree as one of the main reasons for NCSP GPs breaching the contract [34]. In China, the medical master’s degree was conceived as the more established credential than SRT [30]. Physicians with a higher degree are considered to be more competent and may find it easier to secure positions in higher-level hospitals with higher income [30]. Therefore, GPs who are afraid of their failure in gaining admission to the postgraduate studies may break the contract [35]. Indeed, instead of the restriction on obtaining a master’s degree, it might be easier to encourage NCSP GPs to stay by offering them a more community-based, primary-care-oriented postgraduate education [36]. Early and repeated training opportunities in remote communities in the early postgraduate years have been recommended [37]. The education and training curricula could be tailored to fit local contexts [36]. The quality of remote supervision [38]and the exposure to role models during the postgraduate study could also have a positive impact on GPs’ willingness to remain in rural area [39, 40].

In addition, GPs who had a higher score for perception of the completion of six-year obligatory service in rural area were more likely to remain. This is consistent with the result of a medical school in Thailand, in which two-thirds of the graduates continued their rural placements after compulsory training [41]. One possible spill-over effect of NCSP is that GPs may change their work, environment and lifestyle preferences after completing the contract. Though we cannot identify GPs who did not have the intention to stay in rural area before participating in the programme, the GPs who answered “unsure” suggest the possible existence of this unintended effect. The potential advantages of serving in rural area may inspire the graduates to stay when completing the contract, even if they have not had the intention before participating in NCSP. In addition, GPs who scored higher in the perception of not changing designated rural settings were more likely to remain. The restriction was intended to ensure GPs’ fulfilment of the contract and to meet the urgent needs of human resources posed by the designated THCs in rural China. It is possible that GPs who had a good cognition of the value of rural medical work scored high in not changing the designated rural setting as they were inspired to make their own and long-term contributions to the health of rural residents [14].

Our study found that GPs who had a higher perception score on the record in the integrity management were more likely to remain, compared with those who would leave. The integrity management system for health professionals was established in China in 2013 [28], and was used as additional evidence by health institutions to recruit GPs, evaluate their residency performance and help with the decision on GPs’ postgraduate admissions [29]. For GPs who breach the contract and leave, the record may negatively impact their career development, which, could be one explanation for their lower score on this policy compared with GPs who would remain. Accordingly, as one of the penalties for breaching the contract, the integrity management system might be more feasible in ensuring the effectiveness and sustainability of the programme than paying for the fine, if used in an appropriate way.

What’s more, we found that higher scores for completing the SRT and passing the NMLE increases more the risk of GPs leaving rural service compared to the risk of staying. This might be explained by the income differentials between rural and urban practices [42]. GPs who had low valuations of rural life were likely to respond to these income differentials and leave after completing the SRT and passing the NMLE. Their propensity to leave might be even greater if such GPs consider, in addition to wage disparities, postgraduate training restrictions under NCSP and that their demands for career advancement will not be fulfilled in rural service. If so, the clinical knowledge and skills [43] obtained from the SRT and the NMLE will indeed help GPs to leave for more developed urban areas to pursue higher income and better opportunities. In this regard, the completion of SRT and NMLE should not be considered as one of the “incentives” to increase GPs’ intentions to remain. Instead, completing the 3-year SRT and passing NMLE are simply certification requirements by the Chinese government [44, 45] for all GPs to practice medicine and prescribe medications [46]. The aim of these requirements should be to improve qualified abilities of GPs in rural China [12].

For job satisfaction of GPs in our study, the score of satisfaction with income was the lowest among GPs, followed by the score of chances of promotion. The finding aligned well with another conducted among primary care workforce in China who similarly reported low job satisfaction with income level [47]. A previous study showed that 35.5% of 1133 NCSP GPs thought they would plunge into low-income groups once working in the countryside [48]. Indeed, it was possible for primary care workforce, such as village doctors, to earn more than the net per capita income of local residents [49]. However, when compared with counterparts who earned a higher income in urban areas, village doctors were probably discontented with the status quo and preferred to leave [49]. As is investigated in 2017, the median annual income of doctors with a junior professional title (who typically have 2–10 years of clinical experience after graduating from medical college) in central China was 48,000 CNY ($6969), much higher than it was in village clinics of 25,000 CNY ($3630) [42]. In addition, there was an increasement of GPs’ workload to which three tasks related to public health services were added since 2009 [50, 51]. The increased workload extended GPs’ working hours and might further reduce their income from other part-time jobs [52, 53]. Therefore, in order to retain GPs in rural area, the provision of appropriate financial support such as stipends and subsidies should be taken into account [24, 54]. In particular, the access to and a secure of retirement pension has been found to be an important incentive for health professionals to practice in rural settings [31].

The association between chances of promotion and turnover is consistent with the study of primary care doctors in Chongqing, China, which found that chances of promotion significantly predicted turnover intention [26]. Compared with GPs in other countries who focused on job flexibility [55], such as the chance of working in more than one specialty and labour mobility, GPs in China focused more on job stability and employability [56–58]. This difference could be resulted from the relatively new development of primary care and the unclear career prospects of GPs in China [55, 59]. Providing clear career promotion schemes for GPs could potentially address rural doctor shortages and mitigate the influence of turnover on the provision of health care in rural area [60]. The senior posts in rural healthcare settings can help professionals advance their career ladder through experience, education and training, instead of the necessity to leave rural area [61]. The non-pecuniary incentives, such as a stable and safe prospect, the achievement in professional field, the fondness for serving others, and an increased proportion of GPs to obtain national honors and prizes for their outstanding performance are the crucial features of GPs in China that should be taken into account when designing strategies for their career development [62].

Of the GPs surveyed, a large proportion reported low scores for hours of work, the physical working conditions, the freedom of working methods, and the opportunity to use abilities. However, the longer hours GPs work, the less likely they would remain. Indeed, GPs had been playing an expanded role in THCs since the integration of public health services with clinical services in 2009 [63]. The mismatch of higher workload and income contribute to GPs’ low job satisfaction, and may further decrease their intentions to remain in rural area [47, 64]. In addition, GPs who scored higher on the satisfaction with physical working conditions were more likely to remain. In a qualitative study of doctors in rural THCs, working conditions were considered as an important factor of job satisfaction [65]. However, 32 out of 39 participants of the study complained about the disorganized situation in THCs, where limited space was available for squeezing into the many kinds of medicines and diagnostic devices [65]. The lack of air conditioning and sanitation workers were other characteristics of the compromised working conditions of rural THCs in China [65]. To improve the overall conditions of THCs, National Health Commission of China had implemented specific standards on the construction of physical working conditions in THCs in 2022, including the construction area, the number of available beds, and the configuration of public devices. The short and long-term effects of this standard on the job satisfaction and turnover intention among GPs in rural THCs can be studied in future research. The freedom of GPs to make rational clinical decisions and to run their practice [66] were the functional dimensions of autonomy [67]. However, the limitation on drug use might contribute to the decreased autonomy of GPs in China [68]. The decreased freedom of medical practice weakened medical service capacity of GPs to some extent [68], and may further make it difficult for GPs to meet the requirements of their job. For work abilities, one study of GPs in Shanghai found that GPs perceived managerial competence, creativity and entrepreneurship important for their careers [62]. These features should then be considered into the design of job opportunities for GPs to meet their demands-abilities fit [69].

Our study has several limitations. First, the causal interpretation of our results was limited due to the cross-sectional nature of the study. A well conducted, purpose built, ongoing study is recommended to produce robust evidence on the long-term or dynamic effect of perceptions towards the policy and job satisfaction on GPs’ career choice [70]. Second, the survey dataset used in our study is subjected to well-known self-reported bias. For instance, the extent to which rural background predisposes medical students to become rural doctors would be dependent upon some environmental factors about the area [71]. That’s why survey participants might say they are interested in rural or public health service but intentions do not always play out in real-life. Third, additional factors, such as family support and an optimism toward THC development [72] should be identified in future research through in-depth interviews with relevant stakeholders. Fourth, to better modulate the response intensity, a standardized scale such as a 1 to 5 rating system could be used to gauge the intention of stay or leave in the future research. Fifth, considering the contextual differences between provinces, characteristics of rural territories such as the residing populations, the regions’ sizes, the level of urbanization, the capacity of the local primary care service, and the remuneration and payment system should be taken into account in future research to study GPs’ turnover intention.

## Conclusions

The significant associations between GPs’ perceptions towards NCSP, job satisfaction and their intentions to remain suggest important features of GPs for policymakers to take into account when recruiting and retaining rural medical students. Rural healthcare institutions may consider strengthening career advancement of GPs and empowering GPs to build on and use their skills and competencies with the appropriate freedom of choosing their own working methods. Because of the shortage of healthcare workforce in rural area, evidence-based strategies for improving retention in general practice at the primary health care level are needed in China and likely in other settings.

### Supplementary Information

**Additional file 1**. **Table S1**: Characteristics of the 22 provinces that have implemented the NCSP. **Table S2**: NCSP GPs’ intentions to remain in rural area by province.

## Data Availability

The datasets are available from the corresponding author on reasonable request.

## Abbreviations

GPs: General practitioners
NCSP: National Compulsory Service Programme
NCSP GPs: GPs who were enrolled in NCSP
THCs: Township health centres
SRT: Standardised residency training
NMLE: National Medical Licensing Examinations
MEFP: Management of exit from the program
PBC: Penalty for breaching the contract
IST: In-service training
MEFP-1: Restrictions on taking postgraduate exam
MEFP-2: Restrictions on changing designated rural settings
MEFP-3: Commit to work for six years
PBC-1: Pay for the fine
PBC-2: Record in the integrity management
IST-1: Complete the SRT
IST-2: Pass NMLE
RRR: Relative risk ratio
CI: Confidence intervals
