# Supplementary material for: Policy perception, job satisfaction and intentions to remain in rural area: evidence from the National Compulsory Service Programme in China
Source: Glob Health Res Policy. 2024 Apr 30;9:16. doi: 10.1186/s41256-024-00348-z (PMC11059768; doi:10.1186/s41256-024-00348-z)
Supplement: Supplementary file 1 — Additional file 1. Table S1: Characteristics of the 22 provinces that have implemented the NCSP. Table S2: NCSP GPs’ intentions to remain in rural area by province. [file 41256_2024_348_MOESM1_ESM.docx]

Supplementary Material

**Table S1** Characteristics of the 22 provinces that have implemented the NCSP

| **Province** | **Number of GPs enrolled by NCSP** | | | | | **GDP (billion CNY)** | **per capita GDP (CNY)** | **Region ^c^** |
| --- | --- | --- | --- | --- | --- | --- | --- | --- |
|  | **2010-2013** | **2010** | **2011** | **2012** | **2013** |  |  |  |
| Jilin ^a^ | 570 | 180 | 180 | 130 | 80 | 13235.5 | 55,450 | Northeastern |
| Heilongjiang | 650 | 200 | 200 | 140 | 110 | 14879.2 | 47,266 | Northeastern |
| Shanxi ^a^ | 1,080 | 220 | 270 | 270 | 220 | 22590.2 | 64,821 | Central |
| Anhui ^a^ | 695 | 120 | 200 | 195 | 180 | 42959.2 | 70,321 | Central |
| Hubei ^a^ | 800 | 150 | 200 | 200 | 250 | 50012.9 | 86,416 | Central |
| Jiangxi | 1,210 | 260 | 300 | 300 | 350 | 29619.7 | 65,560 | Central |
| Henan | 1,445 | 350 | 350 | 345 | 400 | 58887.4 | 59,410 | Central |
| Hunan | 1,250 | 400 | 300 | 300 | 250 | 46063.1 | 69,440 | Central |
| Guizhou ^a^ | 1,630 | 300 | 350 | 410 | 570 | 19586.4 | 50,808 | Western |
| Guangxi ^a^ | 950 | 200 | 250 | 250 | 250 | 24740.9 | 49,206 | Western |
| Xinjiang ^a^ | 760 | 150 | 200 | 200 | 210 | 15983.6 | 61,725 | Western |
| Qinghai ^a^ | 280 | 70 | 70 | 70 | 70 | 3346.6 | 56,398 | Western |
| Inner Mongolia | 1,095 | 250 | 275 | 270 | 300 | 20514.2 | 85,422 | Western |
| Chongqing | 1,100 | 200 | 300 | 300 | 300 | 27894.0 | 86,879 | Western |
| Sichuan | 2,300 | 500 | 600 | 600 | 600 | 53850.8 | 64,326 | Western |
| Yunnan | 950 | 200 | 250 | 250 | 250 | 27146.8 | 57,686 | Western |
| Tibet | 290 | 80 | 50 | 80 | 80 | 2080.2 | 56,831 | Western |
| Shannxi | 1,070 | 270 | 270 | 265 | 265 | 29801.0 | 75,360 | Western |
| Gansu | 1,130 | 200 | 200 | 310 | 420 | 10243.3 | 41,046 | Western |
| Ningxia | 260 | 50 | 50 | 80 | 80 | 4522.3 | 62,549 | Western |
| Hainan ^a^ | 370 | 80 | 80 | 110 | 100 | 6475.2 | 63,707 | Eastern |
| Hebei | 950 | 320 | 320 | 310 | 0 ^b^ | 40391.3 | 54,172 | Eastern |
| Total GPs | 20,735 | 4,750 | 5,265 | 5,385 | 5,335 | N/A | N/A | N/A |
| GPs of selected provinces  (%) | 7,135 (34%) | 1,470  (31%) | 1,800 (29%) | 1,835  (34%) | 1,930  (36%) | N/A | N/A | N/A |
| Mean value of 22 provinces | N/A | N/A | N/A | N/A | N/A | 25673.8 | 62,945 | N/A |
| Mean value of 9 selected provinces | N/A | N/A | N/A | N/A | N/A | 22103.4 | 62,095 | N/A |

Data Source: The number of GPs enrolled by NCSP of the 22 provinces are sourced from the National Health Commission. GDP and per capita GDP are sourced from China Statistical Yearbook 2021.

Note: ^a^ Provinces selected for our survey.

^b^ Hebei Province has been participating in the NCSP except for the year 2013, as stated by the National Health Commission.

^c^ China Mainland was divided into four economic regions: northeast, east, central, and west.

**Table S2** NCSP GPs’ intentions to remain in rural area by province

| **Province** | **Total**  **N** | **Intentions of remaining in rural area after contract expires**  **N (%)** | | |
| --- | --- | --- | --- | --- |
|  |  | **Remain** | **Leave** | **Unsure** |
| Jilin | 170 | 27 (15.9) | 85 (50.0) | 58 (34.1) |
| Guangxi | 440 | 63 (14.3) | 219 (49.8) | 158 (35.9) |
| Shanxi | 738 | 103 (14.0) | 369 (50.0) | 266 (36.0) |
| Hubei | 175 | 24 (13.7) | 87 (49.7) | 64 (36.6) |
| Hainan | 160 | 20 (12.5) | 61 (38.1) | 79 (49.4) |
| Qinghai | 252 | 31 (12.3) | 134 (53.2) | 87 (34.5) |
| Xinjiang | 299 | 35 (11.7) | 207 (69.2) | 57 (19.1) |
| Guizhou | 958 | 104 (10.9) | 488 (50.9) | 366 (38.2) |
| Anhui | 423 | 35 (8.3) | 257 (60.8) | 131 (31.0) |
| Total | 3,615 | 442 (12.2) | 1,907 (52.8) | 1,266 (35.0) |
